# Supplementary material for: Guidelines for clinical trial protocols for interventions involving artificial intelligence: the SPIRIT-AI extension
Source: Nat Med. 2020 Sep 9;26(9):1351–63. doi: 10.1038/s41591-020-1037-7 (PMC7598944; doi:10.1038/s41591-020-1037-7)
Supplement: Supplementary file 1 — Supplementary Figures 1–2, Supplementary Tables 1–2, Supplementary Note. [file 41591_2020_1037_MOESM1_ESM.pdf]

nature research

https://doi.org/10.1038/s41591-020-1037-7

Supplementary information

Guidelines for clinical trial protocols for interventions involving artificial intelligence: the SPIRIT-AI extension

In the format provided by the authors and unedited

Supplementary Figure 1 (SPIRIT-AI): decision tree for inclusion/exclusion and extension/elaboration.

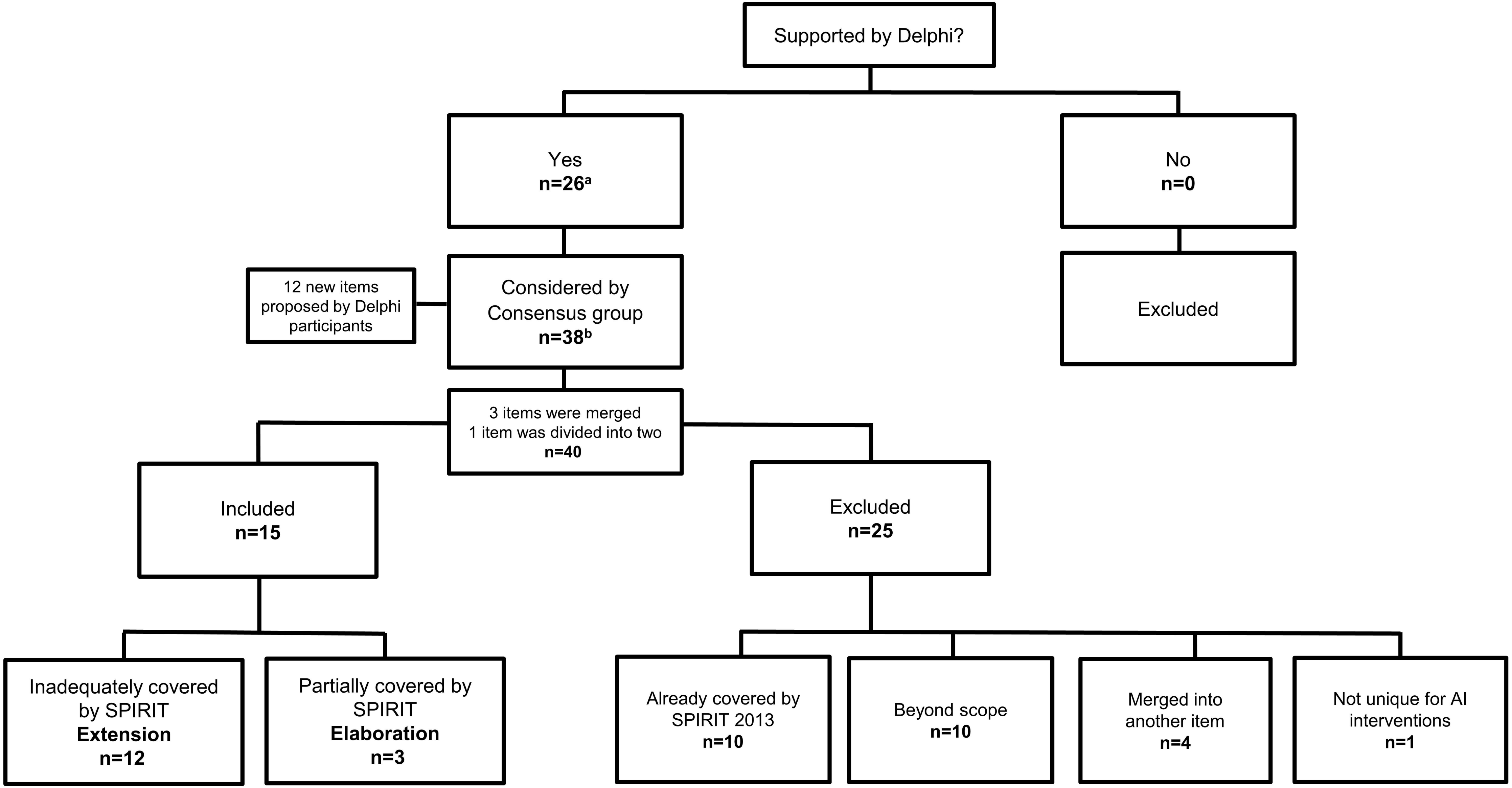

[a] Delphi exercise: inclusion criteria threshold, median score (IQR) ≥4 for (1-3) not important, (4-6) important but not critical and (7-9) important and critical items.
[b] Consensus meeting: inclusion criteria threshold, ≥80% voted included.

Supplementary Figure 2: Checklist Development Process

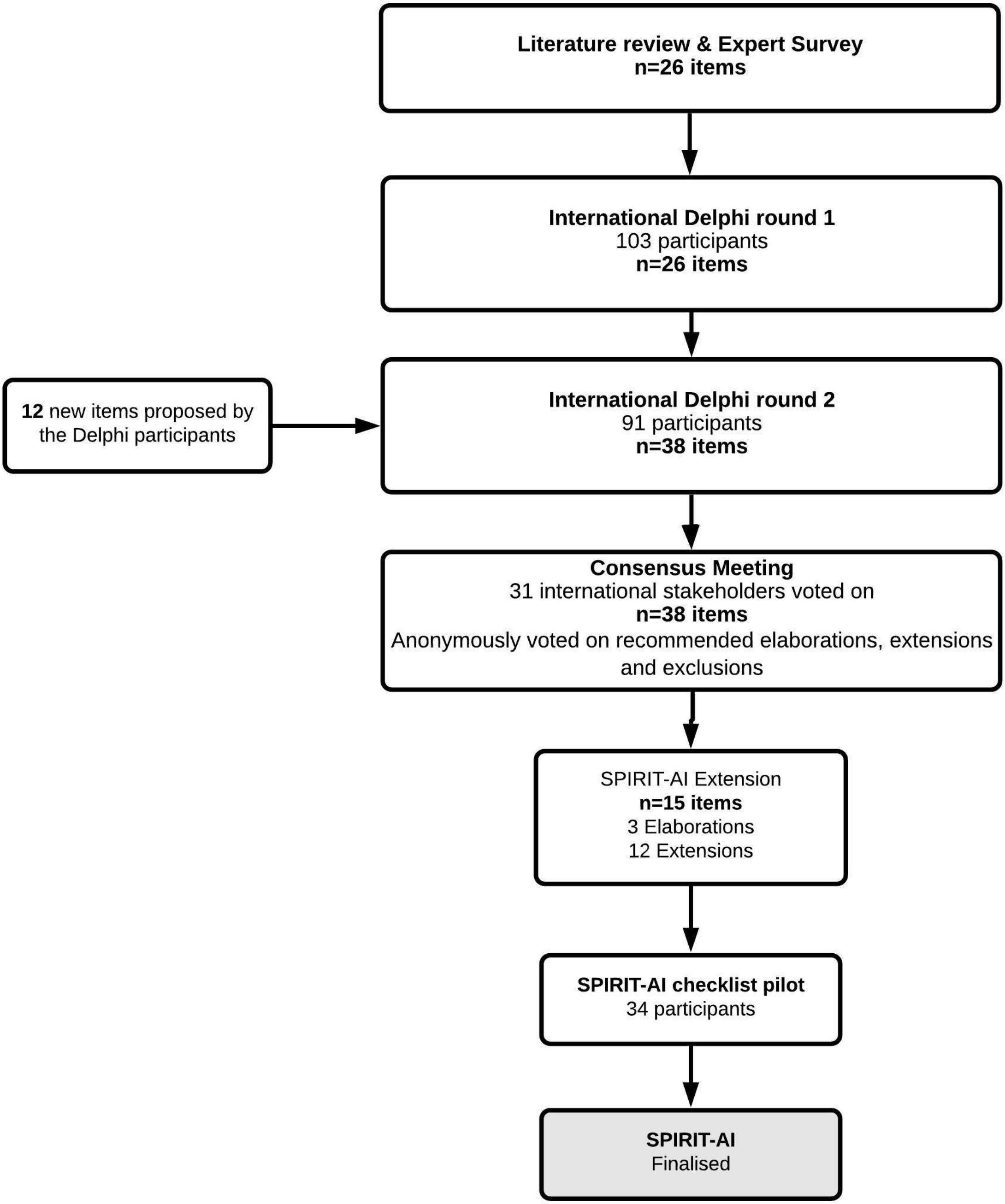

Supplementary Table 1. Characteristics of the Delphi study and consensus meeting participants.

Participants	Delphi survey n=103 (%)	Consensus meeting n=31 (%)
**Area of Expertise**		
Healthcare professional	25(24)	5(16)
Methodologist/Statistician	20(19)	5(16)
Computer scientists	15(14)	3(9)
Industry representatives	11(10)	3(9)
Journal editors	10(9)	6(19)
Policy-makers	6(5)	1(3)
Informatics and healthcare delivery	5(4)	0(-)
Regulators	5(4)	2(6)
Patient advocates	5(4)	3(9)
Funders	4(3)	2(6)
Law and ethics	3(2)	1(3)
Other	14(13)	0(-)
**Experience with clinical trials**		
Trial design	49(47)	11(35)
Trial analysis	57(55)	11(35)
Trial reporting	52(50)	14(45)
Reviewing trials funding	42(40)	10(32)
Research ethics for trials	41(39)	11(35)
Advisory role for policy-makers or commissioning groups for clinical trials	26(25)	5(16)
Some theoretical knowledge but not direct experience	40(38)	7(22)
Additional experience in clinical trials	2(1)	1(3)
**Experience with AI/ML**		
Designing studies to validate AI/ML models	46(44)	11(35)
Developing AI/ML models	47(45)	9(29)
Reviewing AI/ML funding applications	44(42)	9(29)
Implementation of AI/ML in a clinical context	47(45)	7(22)
Some theoretical knowledge on AI/ML but not direct experience	43(41)	12(38)
Advising on transparency and reproducibility of AI/ML models	42(40)	12(38)
Advising on the ethical implications of AI/ML models	31(30)	6(19)
Additional experience in AI	10(9)	3(9)

AI (Artificial intelligence), ML (machine learning)
Participants could select multiple areas of expertise and multiple areas of experience with clinical trials and AI/ML.

Number of participants with expertise in clinical trials and AI/ML: healthcare professionals (n=21); methodologist/statistician (n=18); computer science (n=14); industry representatives (n=5); journal editors (n=9); policy-makers (n=5); informatics and healthcare delivery (n=5); regulators (n=2); patient advocate (n=1); funders (n=1); and law and ethics (n=1).

Supplementary Table 2. Consensus meeting notes and decisions for SPIRIT-AI and CONSORT-AI

	SPIRIT-AI	CONSORT-AI	SPIRIT-AI		CONSORT-AI		SPIRIT-AI	CONSORT-AI				
**Candidate items arising from Delphi Surveys**	**Delphi median score (IQR)**	**Delphi median score (IQR)**	**(%) INCLUDE**	**(%) EXCLUDE**	**(%) INCLUDE**	**(%) EXCLUDE**	**Extension/ Elaboration SPIRIT 2013**	**Extension/ Elaboration CONSORT 2010**	**Reasons for exclusion**	**Consensus meeting discussion notes**	**Final SPIRIT-AI item**	**Final CONSORT-AI item**
Identify the intervention as an AI/machine learning intervention and specify the type of machine learning	8.0 (7.0-9.0)	8.0 (7.0-9.0)	94	6	94	6	**Elaboration**	**Elaboration**		AI may reach broader audience and it might be considered as a more sensitive term. Title should not be too lengthy. AI as opposed to ML may be easier and more accessible for clinicians and systematic reviewers; specification in the abstract. Umbrella term is useful in a situation of evolving terminology. Artificial intelligence and machine learning are useful but the architecture/model is not (consider different training datasets). Regulatory term "medical device". General terms are more useful from a long-term perspective.	**Item 1(i)** Indicate that the intervention involves artificial intelligence/machine learning in the title and/or abstract and specify the type of model	**Item 1a,b (i)** Indicate that the intervention involves artificial intelligence/machine learning in the title and/or abstract and specify the type of model
Specify the purpose of the AI intervention	8.0 (7.0-9.0)	8.0 (7.0-9.0)	90	10	87	13	**Elaboration**	**Elaboration**		Description should be harmonised with regulatory guidance. The specific use should be specified early on, but the intended use can evolve as the technology develops.	**Item 1(ii)** Specify the intended use of the AI intervention	**Item 1a,b (ii)** State the intended use of the AI intervention within the trial in the title and abstract
Describe the intended task of the AI intervention and its interaction with other healthcare professionals	8.0 (7.0-9.0)	7.0 (6.0-8.0)	100	0	100	0	**Extension**	**Extension**	These two items were merged and voted upon as one	Rewording issue: AI-human interface. This item overlaps with the next item and should actually be a subitem. What is the exact role of the AI intervention? What is it compared to? Specify this in the Explanation & Elaboration paper. Include public as well as healthcare processionals as intended users.	**Item 6a (i)** Explain the intended use of the AI intervention in the context of the clinical pathway, including its purpose and its intended users (e.g. healthcare professionals, patients, public).	**Item 2a (i)** Explain the intended use of the AI intervention in the context of the clinical pathway, including its purpose and its intended users (e.g. healthcare professionals, patients, public).
State the intended use of AI intervention in the context of the clinical pathway	8.0 (7.0-9.0)	8.0 (7.0-9.0)								It is important for this point to be accessible by the public; therefore, it should be included in the abstract.		
Describe prior (level) evidence for validation of the AI intervention	7.0 (6.0-8.0)	7.0 (6.0-8.0)	90	10	77	23	**Extension**		For CONSORT, not unique to AI interventions	This item is more suitable for SPIRIT than CONSORT (safety issues). Reword to prior level of validation or feasibility/ level of evidence and provide context for level of evidence. It should be clear if prior validation was for the same use/purpose.	**Item 6a (ii)** Describe any pre-existing evidence for the AI intervention	
Description of the onsite requirements needed to integrate the AI intervention into the trial setting and differences between trials sites	7.0 (6.0-8.0)	6.5 (5.0-8.0)	81	19	83	17	**Extension**	**Extension**		Often you don't know what the implementation process will be. Vital for SPIRIT, but not for CONSORT. Reporting of limitations of the model cloud-based requirements is vital. Minimal requirements is useful to know. From a regulator's perspective, feasibility of implementation is important to know. This item is only relevant if outcomes are key to the infrastructure. There may be major limitations from localisation and replication challenges.	**Item 9** Describe the onsite and offsite requirements needed to integrate the AI intervention into the trial setting.	**Item 4b** Describe how the AI intervention was integrated into the trial setting, including any onsite or offsite requirements.
Describe the inclusion and exclusion criteria at the level of participants; and at the level of the input data	9.0 (8.0 -9.0)	9.0 (8.0-9.0)	100	0	100	0	**Elaboration**	**Elaboration**	Proposed to split into two items (1. participants level 2. input data level) and voted upon both individually	Who and how are vital elements. Example: excluding participants on the basis of imaging quality. Is the quality enough for the algorithm (effectiveness). Input vs participants is not the same thing (i.e. cases vs imaging). It is important to clarify the inclusion and exclusion criteria of the study in order to increase authors understanding. This phase happens before randomization.	**Item 10 (i)** State the inclusion and exclusion criteria at the level of participants.	**Item 4a (i)** State the inclusion and exclusion criteria at the level of participants.
			100	0	100	0	**Extension**	**Extension**			**Item 10 (ii)** State the inclusion and exclusion criteria at the level of input data.	**Item 4 (ii)** State the inclusion and exclusion criteria at the level of input data.
State which version of the AI algorithm is used; if relevant	8.0 (7.0-9.0)	8.0 (7.0-9.0)	90	10	93	7	**Extension**	**Extension**		Important to include the architecture of a deep learning model or include reference of a paper in which details of the algorithm are stated. Version of the AI algorithm is used to compare AI versions over time. This item will need revisiting soon. Include reference to regulatory papers. This item is essential from the regulatory perspective.	**Item 11a (i)** State which version of the AI algorithm will be used.	**Item 5 (i)** State which version of the AI algorithm was used.
Indicate whether the trial setting is the same as the AI intervention development setting	7.0 (6.0-8.0)	7.0 (6.0-8.0)	30	70	26	74			Covered by SPIRIT item 9 and CONSORT item 4b	Any differences in methodology may be important, not exclusively the setting. Difference in performance across sites is common, probably already covered by current guideline but this is important enough for AI that it should be covered again. This item is not specific enough to be relevant. Already covered by CONSORT.		
Describe any interim analyses performed and any changes to the AI intervention	7.0 (7.0-9.0)	7.0 (6.0-9.0)	43	57	53	47			Covered by SPIRIT item 21b and CONSORT item 7b	Useful when you want to adapt the artificial intelligence model within the trial.		
Describe the rationales and assumptions for the sample size calculation	7.0 (6.3-9.0)	7.0 (5.0-9.0)	20	80	16	84			Covered by SPIRIT item 14 and CONSORT item 7a	Core CONSORT/SPIRIT guidelines may cover this already, depending on the trial. It is important to clarify in the Explanation & Elaboration paper.		
Specify sample size calculations carried out to determine reliable control arm intervention			35	65	29	71			Covered by SPIRIT item 14 and CONSORT item 7a	Sample size calculation may be different in artificial intelligence studies (i.e. variability across experts, where experts are the control intervention). This level of variability can have a significant impact on diagnostic validity. Experience of the diagnostician (as the control arm) makes a massive difference to the performance but is this really applicable to an RCT? Important point to include in the elaboration.		
Describe any patient involvement in trial design			58	42	48	52			Beyond scope.	This is not in the original CONSORT and SPIRIT guidelines. Public perception/public awareness is highly stressed, specially in funding applications. This is generic and not AI specific.		

Supplementary Table 2. Consensus meeting notes and decisions for SPIRIT-AI and CONSORT-AI

	SPIRIT-AI	CONSORT-AI	SPIRIT-AI		CONSORT-AI		SPIRIT-AI	CONSORT-AI				
Candidate items arising from Delphi Surveys	Delphi median score (IQR)	Delphi median score (IQR)	(%) INCLUDE	(%) EXCLUDE	(%) INCLUDE	(%) EXCLUDE	Extension/ Elaboration SPIRIT 2013	Extension/ Elaboration CONSORT 2010	Reasons for exclusion	Consensus meeting discussion notes	Final SPIRIT-AI item	Final CONSORT-AI item
Specify any planned ancillary analyses for subgroups where the algorithm is expected to show impaired performance			39	61	35	65			Covered by SPIRIT item 20b and CONSORT item 18	Stratification and subgroup analyses (biases i.e. ethnicity break down) are vital for AI. There are examples of papers which faced criticism because they did not provide substratification for ethnicity. Exploratory analysis is not new for CONSORT or SPIRIT. SPIRIT and CONSORT may cover this already.		
Specify theprotocol for acquiring the input data for the AI intervention	8.0 (5.0-8.0)	7.0 (6.0-9.0)	83	17	84	16	Extension	Extension		How much data cleaning/pre-processing has been done? This is already covered by SPIRIT and CONSORT but it is essential to include it. From the regulatory perspective, this is important for auditing. The version of software should be reported; sufficient information is currently rarely provided. This information is always lacking during the peer review process. This allow you to judge any potential bias and important for replication. Always has to be requested.	**Item 11a (ii)** Specify the procedure for acquiring and selecting the input data for the AI intervention.	**Item 5 (ii)** Describe how the input data were acquired and selected for the AI intervention.
Specify the protocol in the case of missing input data	7.5 (6.0-9.0)	7.0 (7.0-9.0)	73	27	77	23			Revoted upon. Results below.	SPIRIT already covers this item. There are two types of missing data: what goes into the algorithm versus the outcomes in the trial that haven't been collected. This item refers to the part that goes into the algorithm. Pre-specifying the minimal level of quality is vital for safety. Withholding a prediction in case of missing data is also an option. This could be mistakenly confused with the eligibility criteria. Should this refer to the TIDiER checklist? To facilitate reporting, it should be reported in SPIRIT-AI and CONSORT-AI. This is very important as it is one major factor which can be controlled and is the advantage of doing a randomised controlled trial.	**Item 11a (iii)** Specify the procedure for assessing and handling poor quality or unavailable input data.	**Item 5 (iii)** Describe how poor quality or unavailable input data were assessed and handled.
Specify the protocol in the case of missing input data	7.5 (6.0-9.0)	7.0 (7.0-9.0)	97	3	97	3	Extension	Extension				
Specify the protocol for human-AI interaction	7.0 (7.0-9.0)	7.0 (6.0-8.0)	100	0	97	3	Extension	Extension	These two items were merged and voted upon as one.	Only applies if there is an interaction. Specific to the role of the AI intervention. AI-human interaction is critical for ethics comittees. If not defined, there may be down-stream confusion and problems understanding risks. It needs to be clear how management decision was arrived at. This scenario is similar in the case of a genetic test result: managing the consequences of a decision. Interface on the input and output sides are important. Instructions are very important on what to do with a test result. It is recommended to stick to the term "human", as it is broad enough. Helpful to assess risk/benefit ratio for regulators and the public. Specify how the artificial intelligence output is being used to influence decision-making. Patient non-compliance to recommendations also need to be captured.	**Item 11a (iv)** Specify whether there is human-AI interaction in the handling of the input data, and what level of expertise is required of users.	**Item 5 (iv)** Specify whether there was human-AI interaction in the handling of the input data, and what level of expertise was required of users.
Detail the required level of expertise of health care professionals and operators for interacting with the AI intervention	6.0 (5.0-7.5)	6.0 (5.0-7.0)										
Specify what is the output of the AI intervention	9.0 (8.0-9.0)	9.0 (7.0-9.0)	100	0	100	0	Extension	Extension		There was no discussion around this item. Consensus participants decided to go straight to voting because of the high importance of the item.	**Item 11a (v)** Specify the output of the AI intervention.	**Item 5 (v)** Specify the output of the AI intervention.
Explain the protocol for how the AI intervention will lead to treatment decision-making	8.0 (6.0-8.0)	8.0 (7.0-9.0)	94	6	97	3	Extension	Extension		Actual regulatory decision. Treatment decision may not be done by the person that has interacted with the artificial intelligence intervention. Take out 'treatment' and make the decision making element more broad.	**Item 11a (vi)** Explain the procedure for how the AI intervention's outputs will contribute to decision-making or other elements of clinical practice.	**Item 5 (vi)** Explain how the AI intervention's outputs contributed to decision-making or other elements of clinical practice.
Describe the control intervention sufficient to allow replication			20	80	20	80			Covered by SPIRIT item 11a and CONSORT item 5	Control arms tend to be poorly reported (often just describer as "usual care"). Mandating for what is reported in the interventional arm should be reported in the control arm. It may be very expensive to get all this information (not necessarily mandatory). This item is already covered by SPIRIT and CONSORT, captured in 'description for intervention'.		
Provide an explanation of how uncertainty from the intervention will be communicated to end users			17	83	10	90			Beyond scope	Explainability can lead to uncertainy and introduce bias. First read it as a technical requirement. Uncertainty: concerns related to communication can introduce bias in certain ways.		
State whether the AI algorithm is a static model; or if it is continuously evolving. If the latter; provide details	9.0 (7.0-9.0)	9.0 (7.0-9.0)	30	70	27	73			These two items were merged and voted upon together. Beyond scope.	This is an evolving field and we are lacking tangible examples of continuously updating medical AI algorithms to provide guidance on this item. Technology is not ready yet, therefore we cannot provide any guidance. There's a huge danger of overfitting. It is important to have an item where we specify the algorithm version/ architecture/ evolving vs static (to be included in Explanation & Elaboration). Important to revisit SPIRIT-AI and CONSORT-AI in a few years.		
Describe the nature of continuous updating of the AI intervention; if relevant	9.0 (7.0-9.0)	9.0 (7.0-9.0)										
Describe the type of model and/or reference details of the AI algorithm			77	23	74	26			Beyond scope	(New item generated during consensus meeting discussion and voted upon)		
In the case of continuously updating algorithms, describe the new training data									Beyond scope	Beyond scope		

Supplementary Table 2. Consensus meeting notes and decisions for SPIRIT-AI and CONSORT-AI

	SPIRIT-AI	CONSORT-AI	SPIRIT-AI		CONSORT-AI		SPIRIT-AI	CONSORT-AI				
Candidate items arising from Delphi Surveys	Delphi median score (IQR)	Delphi median score (IQR)	(%) INCLUDE	(%) EXCLUDE	(%) INCLUDE	(%) EXCLUDE	Extension/ Elaboration SPIRIT 2013	Extension/ Elaboration CONSORT 2010	Reasons for exclusion	Consensus meeting discussion notes	Final SPIRIT-AI item	Final CONSORT-AI item
In the case of continuously updating algorithms, report the level at which the data was partitioned for training and for validation/testing									Beyond scope	Beyond scope		
State any deviations from trial protocol			NA	NA	52	48			Not unique to AI interventions	It is good to be transperent about the deviations. This is currently not captured but will be when the SPIRIT-AI and CONSORT-AI are revised in the future. Add examples. Link to regulatory guidance in jurisdiction.		
Report instances of misuse of the AI intervention recommendations; if relevant	7.0 (5.0-7.0)	7.0 (6.0-9.0)	45	55	47	53			Beyond scope	How would people report that? Analogous to cross-over/ intervention that wasn't used in the way it was intended. Misused against intended use. From the regulatory perspective, it is important to state the reason why the AI intervention was misused. In a report this will be important (incidences/adverse events). This item is already covered by SPIRIT. This is not specific to artificial intelligence. It is important to know why something wasn't adhered to.		
Describe the procedures and any occurrences of data breach	7.0 (6.0-9.0)	7.0 (6.0-9.0)	32	68	26	74			Covered by SPIRIT item 22 and CONSORT item 19	This item does not seem to differ from SPIRIT or CONSORT. SPIRIT: procedures in the event of any data breach. CONSORT: any occurences of data breach.		
Where the AI intervention is a diagnostic or predictive model, provide a detailed summary of the false positives and false negatives<br><br>Describe anticipated undesirable outcomes and risks, including worst-case scenario			87	13	90	10	Extension	Extension	These two items were merged and voted upon together	Error analysis is vital (i.e. substratification due to ethnicity). This applies in the case of re-training due to systematic error (accuracy as part of the trial). Posthoc analysis is vital - people behaving unpredictable in each arm can be scrutinised. Identify subgroups in which the artificial intelligence should not be deployed in order to identify all errors and risk mitigation strategy vital.	**Item 22** Specify any plans to identify and analyse performance errors. If there are no plans for this, justify why not.	**Item 19** Describe results of any performance errors and how were identified, where applicable. If no such analysis was planned or done, justify why not.
Use of AI should be explicitly described in consent materials	8.0 (6.0-9.0)	7.0 (5.0-9.0)	27	73	21	79			Beyond scope	This item is not unique to artificial intelligence. Ethics panel should decide whether artificial intelligence should be explicity described in the participant consent form.		
State whether participant data can be safely withdrawn from the clinical trial, if needed			3	97	NA	NA			Beyond scope	Data can not be fully withdrawn and should be mentioned in the participant consent form. This item is not unique to artificial intelligence.		
Interpret results in the context of differences between the dataset used to develop and validate the AI intervention and the clinical trial data	NA	7.0 (6.0-9.0)			21	79			Covered CONSORT Item 21	Artificial intelligence is specific in the sense that the intervention can be improved with every intervention. However, CONSORT already covers this item. Not necessarily unique to AI. Provide minimum list of things to report and examples of types of biases.		
Explain the underlying assumptions and mechanisms of the AI intervention and uncertainties of the results	NA	8.0 (6.0-9.0)			19	81			Covered by CONSORT item 20	Mandate some a priori analyses. Combine generalisability/bias analyses: input data, population and setting. This point is not about generalisability, which would happen in the future. This point is about pre-validation. Authors will likely explain under performance. This item is unique and it complements the point on versioning of the algorithm.		
Describe potential biases stemming from the included participants/data	NA	8.0 (7.0-9.0)			48	52			Covered by CONSORT item 20	Regulators want to know what devices/softwares were used. Important to include this in the Exaplanation & Elaboration paper. Minimum list of things that should be reported and examples of types of biases.		
If applicable; plans for any attempts to audit; decode or explain the AI intervention's recommendations	6.0 (6.0-9.0)	6.0 (5.0-9.0)	60	40	47	53			Covered by SPIRIT item 20b CONSORT item 22	Important to identify biases of the dataset. Interpretability may be harmful in certain cases. Currently explainability methods are not understandable in a straight forward way, however this is an issue that is unique to AI. Prespecification is vital. This should be done at an earlier stage - i.e. before the clinical trials stage. Explainability can inappropiately confer trust. Some situations where explainability is more tractable. Authors should state they will do it, but unreasonable to ask for prespecified analysis. Not to be seen as endorsing something that is unclear.		
Availability of the AI Intervention Code	7.0 (5.0-9.0)	7.0 (5.0-9.0)	100	0	100	0	Extension	Extension		It is important to release the architecture code and parameters for transparency purposes. Data sharing is useless without the coding. Funders perspective: it is important to share the code, specially if funded so it can be used/replicated. Availability of the the coding doesn't mean the AI model would be easy to replicate. It should be stated if the coding is available and under what license. Important to mandate commercially availabilty: which regulator approved it, unique identifier and which class. Not unique to AI. This item is not advocating for code sharing, but rather just to declare whether code is available.	**Item 29** State whether and how the AI intervention and/or its code can be accessed, including any restrictions to access or re-use.	**Item 25** State whether and how the AI intervention and/or its code can be accessed, including any restrictions to access or re-use.
Patents and patent applications for the AI intervention	6.0 (6.0-8.0)	6.0 (5.0-7.0)	7	93	7	93			Beyond scope			

Supplementary Table 2. Consensus meeting notes and decisions for SPIRIT-AI and CONSORT-AI

	SPIRIT-AI	CONSORT-AI	SPIRIT-AI		CONSORT-AI		SPIRIT-AI	CONSORT-AI				
**Candidate items arising from Delphi Surveys**	**Delphi median score (IQR)**	**Delphi median score (IQR)**	**(%) INCLUDE**	**(%) EXCLUDE**	**(%) INCLUDE**	**(%) EXCLUDE**	**Extension/ Elaboration SPIRIT 2013**	**Extension/ Elaboration CONSORT 2010**	**Reasons for exclusion**	**Consensus meeting discussion notes**	**Final SPIRIT-AI item**	**Final CONSORT-AI item**
Role of the AI developer	6.0 (6.0-8.0)	6.0 (5.0-7.0)	3	97	0	100			Covered by SPIRIT item 28 CONSORT item 25	This item already covered by SPIRIT, authorship section. In addtition, the item is not unique to artificial intelligence.		
Describe the role of the sponsor			0	100	0	100			Covered by SPIRIT item 28 CONSORT item 25	It is not an artificial intelligence specific item. It is already covered by existing guidance.		

Supplementary Note

The SPIRIT-AI and CONSORT-AI Group gratefully acknowledge the contributions of the participants of the Delphi study and for providing feedback through final piloting of the checklist.

Delphi study participants: Aaron Y. Lee (Department of Ophthalmology, University of Washington, Seattle, WA, USA), Adrian Jonas (The National Institute for Health and Care Excellence (NICE), London, UK), Alastair K. Denniston (Academic Unit of Ophthalmology, Institute of Inflammation and Ageing, University of Birmingham, Birmingham, UK; University Hospitals Birmingham NHS Foundation Trust, Birmingham, UK; Health Data Research UK, London, UK; Centre for Patient Reported Outcomes Research, Institute of Applied Health Research, University of Birmingham, Birmingham, UK), Andre Esteva (Salesforce Research, San Francisco, CA, USA), Andrew Beam (Harvard T.H. Chan School of Public Health, Boston, MA, USA), Andrew Goddard (Royal College of Physicians, London, UK), Anna Koroleva (Universite Paris-Saclay, Orsay, France and Academic Medical Center, University of Amsterdam, Amsterdam, the Netherlands), Annabelle Cumyn (Department of Medicine, Université de Sherbrooke, Quebec, Canada), Anuj Pareek (Center for Artificial Intelligence in Medicine & Imaging, Stanford University, CA, USA), An-Wen Chan (Department of Medicine, Women's College Research Institute, Women's College Hospital, University of Toronto, Ontario, Canada), Ari Ercole (University of Cambridge, Cambridge, UK), Balaraman Ravindran (Indian Institute of Technology Madras, Chennai, India), Bu'Hassain Hayee (King's College Hospital NHS Foundation Trust, London, UK), Camilla Fleetcroft (Medicines and Healthcare products Regulatory Agency, London, UK), Cecilia Lee (Department of Ophthalmology, University of Washington, Seattle, WA, USA), Charles Onu (Mila - the Québec AI Institute, McGill University and Ubenwa Health, Montreal, Canada), Christopher Holmes (Alan Turing Institute, London, UK), Christopher Kelly (Google Health, London, UK), Christopher Yau (University of Manchester, Manchester, UK; Alan Turing Institute, London, UK), Cynthia D. Mulrow (Annals of Internal Medicine, Philadelphia, PA, USA), Constantine Gatsoins (Brown University, Providence, RI, USA), Cyrus Espinoza (Patient Partner, Birmingham, UK), Daniela Ferrara (Tufts University, Medford, MA, USA), David Moher (Centre for Journalology, Clinical Epidemiology Program, Ottawa Hospital Research Institute, Ottawa, Canada), David Watson (Green Templeton College, University of Oxford, Oxford, UK), David Westhead (School of Molecular and Cellular Biology, University of Leeds, Leeds, UK), Deborah Morrison (National Institute for Health and Care Excellence (NICE), London, UK), Dominic Danks (Institute of Cancer and Genomic

Sciences, University of Birmingham, Birmingham, UK and The Alan Turing Institute, London, UK), Dun Jack Fu (Moorfields Hospital London NHS Foundation Trust, London, UK), Elaine Manna (Patient Partner, London, UK), Eric Rubin (New England Journal of Medicine, Boston, MA, USA), Ewout Steyerberg (Leiden University Medical Centre and Erasmus MC, Rotterdam, the Netherlands), Fiona Gilbert (University of Cambridge and Addenbrooke's Hospital, Cambridge, Cambridge, UK), Frank E Harrell Jr, (Department of Biostatistics, Vanderbilt University School of Medicine, Nashville, TN, USA), Gary Collins (Centre for Statistics in Medicine, University of Oxford, Oxford, UK), Gary Price (Patient Partner, Centre for Patient Reported Outcome Research, Institute of Applied Health Research, University of Birmingham, Birmingham, UK), Giovanni Montesano (City, University of London - Optometry and Visual Sciences, London, UK; NIHR Biomedical Research Centre, Moorfields Eye Hospital NHS Foundation Trust and UCL Institute of Ophthalmology, London, UK), Hannah Murfet (Microsoft Research Ltd, Cambridge, UK), Heather Mattie (Harvard T.H. Chan School of Public Health, Harvard University, Boston, MA, USA), Henry Hoffman (Ada Health GmbH, Berlin, Germany), Hugh Harvey (Hardian Health, London, UK), Ibrahim Habli (Department of Computer Science, University of York, York, UK), Immaculate Motsi-Omoijiade (Business School, University of Birmingham, Birmingham, UK), Indra Joshi (Artificial Intelligence Unit, National Health Service X (NHSX), UK), Issac S. Kohane (Harvard University, Boston, MA, USA), Jeremie F. Cohen (Necker Hospital for Sick Children, Université de Paris, CRESS, INSERM, Paris, France), Javier Carmona (Nature Research, New York, NY, USA), Jeffrey Drazen (New England Journal of Medicine, MA, USA), Jessica Morley (Digital Ethics Lab, University of Oxford, Oxford, UK), Joanne Holden (National Institute for Health and Care Excellence (NICE), Manchester, UK), Joao Monteiro (Nature Research, New York, NY, USA), Joseph R. Ledsam (DeepMind Technologies, London, UK), Karen Yeung (Birmingham Law School, University of Birmingham, Birmingham, UK), Karla Diaz Ordaz (London School of Hygiene and Tropical Medicine and Alan Turing Institute, London, UK), Katherine McAllister (Health and Social Care Data and Analytics, National Institute for Health and Care Excellence (NICE), London, UK), Lavinia Ferrante di Ruffano (Institute of Applied Health Research,University of Birmingham, Birmingham, UK), Les Irwing (Sydney School of Public Health, University of Sydney, Sydney, Australia), Livia Fas (Medical Retina Department, Moorfields Eye Hospital NHS Foundation Trust, London, UK and Eye Clinic, Cantonal Hospital of Lucerne, Lucerne, Switzerland), Luke Oakden-Rayner (Australian Institute for Machine Learning, North Terrace, Adelaide, Australia), Marcus Ong (Spectra Analytics, London ,UK), Mark Kelson (The Alan Turing Institute, London, UK and University of Exeter, Exeter, UK), Mark Ratnarajah (C2-AI, Cambridge, UK), Martin Landray (Nuffield Department of Population Health, University of Oxford, Oxford, UK), Masashi Misawa (Digestive Disease Center, Showa University, Northern Yokohama Hospital, Yokohama,

Japan), Matthew Fenech (Ada Health GmbH, Berlin, Germany), Maurizio Vecchione (Intellectual Ventures, Bellevue, WA, USA), Megan Wilson (Google Health, London, UK), Melanie J. Calvert (Centre for Patient Reported Outcomes Research, Institute of Applied Health Research, University of Birmingham, Birmingham, UK; National Institute of Health Research Surgical Reconstruction and Microbiology Centre, University of Birmingham and University Hospitals Birmingham NHS Foundation Trust, Birmingham, UK; National Institute of Health Research Applied Research Collaborative West Midlands), Michel Vaillant (Luxembourg Institute of Health, Luxembourg), Nico Riedel (Berlin Institute of Health, Berlin, Germany), Niel Ebenezer (Fight for Sight, London, UK), Omer F Ahmad (Wellcome/EPSRC Centre for Interventional & Surgical Sciences, University College London, London, UK), Patrick M. Bossuyt (Department of Clinical Epidemiology, Biostatistics and Bioinformatics, Amsterdam University Medical Centers, the Netherlands), Pep Pamies (Nature Research, London, UK), Philip Hines (European Medicines Agency (EMA), Amsterdam, the Netherlands), Po-Hsuan Cameron Chen (Google Health, Palo Alto, CA, USA), Robert Golub (Journal of the American Medical Association, The JAMA Network, Chicago, IL, USA), Robert Willans (National Institute for Health and Care Excellence (NICE), Manchester, UK), Roberto Salgado (Department of Pathology, GZA-ZNA Hospitals, Antwerp, Belgium and Division of Research, Peter Mac Callum Cancer Center, Melbourne, Australia), Ruby Bains (Gastrointestinal Diseases Department, Medtronic, UK), Rupa Sarkar (Lancet Digital Health, London, UK), Samuel Rowley (Medical Research Council (UKRI), London, UK), Sebastian Zeki (Department of Gastroenterology, Guy's and St Thomas' NHS Foundation Trust, London, UK), Siegfried Wagner (NIHR Biomedical Research Centre at Moorfields Eye Hospital and UCL Institute of Ophthalmology, London, UK), Steve Harries (Institutional Research Information Service, University College London, London, UK), Tessa Cook (Hospital of University of Pennsylvania, Pennsylvania, PA, USA), Trishan Panch (Wellframe, Boston, MA, USA), Will Navaie (Health Research Authority (HRA), London, UK), Wim Weber (British Medical Journal, London, UK), Xiaoxuan Liu (Academic Unit of Ophthalmology, Institute of Inflammation and Ageing, University of Birmingham, Birmingham, UK; University Hospitals Birmingham NHS Foundation Trust, Birmingham, UK; Halth Data Research UK, London, UK), Yemisi Takwoingi (Institute of Applied Health Research, University of Birmingham, Birmingham, UK), Yuichi Mori (Digestive Disease Center, Showa University, Northern Yokohama Hospital, Yokohama, Japan), Yun Liu (Google Health, Palo Alto, CA, USA).

Pilot study participants: Andrew Marshall (Nature Research, New York, NY, USA), Anna Koroleva (Universite Paris-Saclay, Orsay, France and Academic Medical Center, University of Amsterdam, Amsterdam, the Netherlands), Annabelle Cumyn (Department of Medicine,

Université de Sherbrooke, Quebec, Canada), Anna Goldenberg (SickKids Research Institute, Toronto, ON, Canada), Anuj Pareek (Center for Artificial Intelligence in Medicine & Imaging, Stanford University, CA, USA), Ari Ercole (University of Cambridge, Cambridge, UK), Ben Glocker (BioMedIA, Imperial College London, London, UK), Camilla Fleetcroft (Medicines and Healthcare products Regulatory Agency, London, UK), David Westhead (School of Molecular and Cellular Biology, University of Leeds, Leeds, UK), Eric Topol (Scripps Research Translational Institute, La Jolla, CA, USA), Frank E. Harrell Jr, (Department of Biostatistics, Vanderbilt University School of Medicine, Nashville, TN, USA), Hannah Murfet (Microsoft Research Ltd, Cambridge, UK), Ibarahim Habli (Department of Computer Science, University of York, York, UK), Jeremie F. Cohen (Necker Hospital for Sick Children, Université de Paris, CRESS, INSERM, Paris, France), Joanne Holden (National Institute for Health and Care Excellence (NICE), Manchester, UK), John Fletcher (British Medical Journal, London, UK), Joao Monteiro (Nature Research, New York, NY, USA), Joseph R. Ledsam (DeepMind Technologies, London, UK), Mark Ratnarajah (C2-AI, London, UK), Matthew Fenech (Ada Health GmbH, Berlin, Germany), Michel Vaillant (Luxembourg Institute of Health, Luxembourg), Omer F. Ahmad (Wellcome/EPSRC Centre for Interventional & Surgical Sciences, University College London, London, UK), Pep Pamies (Nature Research, London, UK), Po-Hsuan Cameron Chen (Google Health, Palo Alto, CA, USA), Robert Golub (Journal of the American Medical Association, The JAMA Network, Chicago, IL, USA), Roberto Salgado (Department of Pathology, GZA-ZNA Hospitals, Antwerp, Belgium and Division of Research, Peter Mac Callum Cancer Center, Melbourne, Australia), Rupa Sarkar (Lancet Digital Health, London, UK), Siegfried Wagner (Ophthalmology, Moorfields Eye Hospital NHS Foundation Trust, London, UK), Suchi Saria (Johns Hopkins University, Baltimore, MD, USA), Tessa Cook (Hospital of University of Pennsylvania, Pennsylvania, PA, USA), Thomas Debray (University Medical Center Utrecht, Utrecht, the Netherlands), Tyler Berzin (Beth Israel Deaconess Medical Center and Harvard Medical School, Boston, MA, USA), Wanda Layman (Nature Research, New York, NY, USA), Wim Weber (British Medical Journal, London, UK), Yun Liu (Google Health, Palo Alto, CA, USA).
